# A DeepSeek cross-modal platform for personalized art education in Autism Spectrum Disorder

**DOI:** 10.1038/s41598-025-28518-0

**Published:** 2025-12-29

**Authors:** Yaoyao Ding, Zichang Li, Yuntao Zou, Xiao Dong

**Affiliations:** 1 School of Design and Art, Communication University of Zhejiang, Hangzhou, China; 2 Future Front Interdisciplinary Research Institute, Huazhong University of Science and Technology, Wuhan, China; 3 Faculty of Art and Design, KEYI College of Zhejiang Sci University, Hangzhou, China; 4 Advanced Interdisciplinary Research Center, City University of Macau, Macao, China; 5 School of Arts and Media, Hubei University of Commerce and Trade, Wuhan, China

**Keywords:** Educational equity, Autism spectrum disorder, AI-assisted learning, Inclusive art education, Sensory accessibility, Neurodivergent education, Cross-modal integration, Digital inclusion, Computer science, Psychology

## Abstract

Educational inequity in arts learning disproportionately marginalizes students with Autism Spectrum Disorder (ASD), who require structured, predictable environments for aesthetic development and sensory regulation that traditional pedagogies fail to provide. This study introduces an AI-powered e-learning platform that addresses these systematic barriers through intelligent cross-modal integration, democratizing access to personalized art education for neurodivergent learners. Our DeepSeek-based system transforms visual art features into structured musical accompaniments that accommodate individual sensory processing patterns, cultural backgrounds, and neurodevelopmental profiles while maintaining the predictability essential for ASD learning success. The platform employs enhanced ResNet-50 architecture, high-dimensional manifold mapping, and conditional generation models specifically optimized for sensory regulation principles. Comprehensive evaluation with 203 participants (including 53 neurodivergent learners) and 19 autism education specialists demonstrates substantial improvements: sensory comfort ratings of 4.6/5, learning satisfaction of 4.3/5, and educational outcomes showing 20.5% NAEP score improvements compared to 8.2% for traditional methods (p < 0.008). Technical performance achieved superior cross-modal consistency (MSE 0.05, PCC 0.92) with 89% accommodation success across diverse sensory profiles. This research offers a promising model for inclusive digital education by demonstrating how AI can contribute to mitigating educational inequities for neurodivergent populations. It provides a scalable framework that advances accessible arts education, embracing neurodiversity while maintaining academic rigor.

## Introduction

The profound disparities in educational opportunities for neurodivergent learners represent one of the most pressing equity challenges in contemporary education. Framed through the lens of the social model of disability, these challenges are understood not as deficits inherent to the learner, but as systemic barriers constructed by inflexible educational environments^1,2^. In art education, traditional pedagogy—with its reliance on open-ended social collaboration and a singular mode of sensory engagement—creates such a “disabling environment” for many students with Autism Spectrum Disorder (ASD). For these learners, who often thrive with structure and predictability, the conventional art classroom can become a site of sensory overwhelm and cognitive friction^3^, leading to their systematic marginalization from creative expression and perpetuating educational inequity^4,5^.

Addressing these environmental barriers requires a pedagogical shift towards inclusive frameworks, chief among them the principles of Universal Design for Learning (UDL). The UDL framework posits that to create truly equitable learning opportunities, curricula should be designed from the outset to accommodate learner variability by providing multiple means of representation, engagement, and expression^6,7^. For arts education, this implies moving beyond a purely visual-spatial modality and offering alternative pathways for aesthetic appreciation and understanding^8^. By providing multiple, structured ways to access the core concepts of an artwork, a UDL-aligned approach can dismantle the very barriers that marginalize neurodivergent students, transforming the learning environment from one of exclusion to one of genuine access.

While the UDL framework provides a powerful pedagogical blueprint, its practical implementation in a domain as nuanced as the visual arts presents significant challenges. The semantic richness of artworks—encompassing composition, color theory, cultural context, and emotional resonance—resists straightforward translation into alternative modalities. Moreover, effective UDL implementation requires not merely a one-size-fits-all conversion, but individualized adaptations responsive to each learner’s unique sensory profile and cognitive needs^9,10^. Manually creating such personalized, multi-modal learning materials for diverse artworks is resource-intensive and often beyond the practical reach of most educators, particularly in under-resourced settings^11,12^.

Recent advances in large language models (LLMs), particularly systems like DeepSeek, offer unprecedented opportunities to address educational equity challenges in autism-specific art education^13–16^. LLMs can democratize access to sophisticated pedagogical content by enabling non-technical educators to create personalized, structured learning experiences that accommodate diverse sensory and cognitive needs^17,18^. Through semantic analysis and contextual modeling, these systems can transform static visual artworks into predictable, multi-sensory educational experiences that align with ASD learners’ preference for clear structure and sensory regulation^19–21^. Crucially, LLMs enable educators without technical expertise to operationalize the principles of UDL at scale, thereby democratizing access to inclusive arts education and offering a tangible pathway toward transforming the disabling environment into one of genuine educational equity^22,23^. Such capabilities position LLMs as powerful tools for creating inclusive educational environments that respect neurodiversity while maintaining pedagogical rigor.

This study introduces an AI-driven e-learning platform specifically designed to enhance educational equity for ASD students through personalized music-assisted art education. The platform leverages DeepSeek’s capabilities to integrate visual features from paintings with carefully selected auditory elements, creating structured multi-sensory learning experiences that accommodate ASD-specific cognitive patterns and sensory preferences^24,25^. Unlike conventional approaches that rely on unpredictable sensory presentations, our system transforms visual aesthetic elements into systematic keywords and maps these to music-specific attributes that support sustained attention, emotional regulation, and sensory comfort. By incorporating individual learner profiles—including sensory sensitivities, communication preferences, and behavioral patterns—the platform delivers tailored educational experiences that promote both artistic understanding and cognitive accessibility within predictable frameworks.

The platform’s technical framework employs high-dimensional manifold mapping and cross-modal alignment techniques to ensure coherent integration of visual and auditory modalities while maintaining sensory predictability^26^. Conditional generative models, optimized through continuous user feedback and sensory monitoring, enable real-time adaptation to individual learning needs and sensory thresholds while maintaining accessibility for educators without technical expertise. Through comprehensive evaluation with ASD students and autism education professionals, we validate the platform’s effectiveness in improving engagement, learning outcomes, sensory comfort, and educational satisfaction, demonstrating its potential to address systemic inequities in autism-inclusive art education.

This work contributes a scalable, evidence-based solution that directly addresses educational equity challenges for autistic learners through a neurodiversity-affirming approach^27^. By integrating visual and auditory arts within a sensorially accessible and structurally predictable framework, it offers a technology-driven pathway for inclusive digital art education that aligns with contemporary autism education goals while supporting broader neurodiversity acceptance initiatives in educational settings.

## Literature review

### Pedagogical foundations for inclusive arts education

Contemporary disability scholarship challenges traditional medicalized frameworks that locate learning difficulties within individual pathologies, instead positioning disability as a product of environmental and systemic barriers^28,29^. This perspective, rooted in the social model of disability, directs critical attention toward the design of learning environments themselves as sites of exclusion or inclusion^30^. In art education specifically, conventional pedagogical approaches—predicated on neurotypical assumptions about sensory processing and creative expression—inadvertently construct such “disabling environments” for many neurodivergent learners^23,31^. For students with ASD, the traditional art classroom often presents multiple intersecting barriers, from unpredictable sensory stimuli to pedagogical methods that provide insufficient structure for learners who benefit from predictable routines^32^. These environmental features, rather than inherent deficits, constitute the primary obstacles to meaningful participation in arts education.

Universal Design for Learning (UDL) has emerged as a comprehensive pedagogical framework explicitly aligned with the social model’s emphasis on environmental modification^6,7^. Grounded in neuroscience, UDL proposes that curricula should accommodate diverse learners from the outset by providing multiple means of representation, action and expression, and engagement^33^. For arts education, the principle of multiple means of representation holds particular significance, as traditional instruction privileges visual-spatial processing through a single sensory modality. A UDL-aligned approach would necessarily provide alternative pathways for engaging with artworks, thereby dismantling the sensory monoculture that marginalizes neurodivergent learners. Systematic reviews confirm the effectiveness of UDL in enhancing educational access and outcomes, operationalizing the social model’s commitment to transforming disabling environments into genuinely inclusive learning spaces^34^.

However, a significant gap persists between the pedagogical promise of UDL and its widespread implementation in arts education^9,35^. The semantic complexity of visual artworks resists straightforward conversion into alternative modalities, requiring sophisticated interpretation. Moreover, effective UDL demands individualized accommodations responsive to each learner’s unique sensory profile, multiplying the complexity for educators^36^. Systematic reviews of UDL implementation reveal persistent barriers, including insufficient institutional resources, time constraints, and a lack of accessible tools for generating high-quality multimodal learning materials^37^. This implementation gap establishes the critical need for scalable technological solutions that can bridge theory and practice, empowering educators to operationalize UDL principles in real-world classroom settings^12^.

### Educational equity and technology support for ASD learners

Autism Spectrum Disorder affects approximately 1–2% of school-age children globally, creating significant educational equity challenges that traditional pedagogical approaches struggle to address effectively. Students with ASD face systematic barriers in conventional educational settings, particularly in subjects requiring social interaction and sensory flexibility, such as art education. These learners often demonstrate superior outcomes when educational content is delivered through structured, predictable frameworks that accommodate their unique sensory processing patterns and communication preferences, yet most current educational technologies fail to provide such accommodations.

Large language models have emerged as transformative tools with significant potential to address educational equity challenges through their advanced natural language processing and knowledge integration capabilities^38^. However, research specifically targeting neurodivergent populations remains limited, with most applications focusing on general educational contexts rather than the specialized needs of ASD learners. This represents a critical gap in educational technology development, as effective support for autistic students requires approaches that go beyond general accessibility improvements to address specific sensory processing, communication, and structural learning needs.

Initial explorations have demonstrated promise but reveal significant limitations. Giretti et al. investigated LLM integration in art education through systems like BLOOM, showing potential for supporting diverse cognitive processing patterns^39^. Lee et al. developed LLaVA-Docent, a multimodal system for art appreciation that suggests possibilities for structured learning approaches beneficial to ASD students^40^. Zhang et al. explored collaborative applications that could support students with varied communication strengths^41^. Despite these advances, current systems lack the specific design principles necessary for supporting sensory regulation and structured learning preferences characteristic of ASD populations.

The therapeutic potential of technology-enhanced learning remains largely untapped for ASD populations. Chakrabarty et al. noted that while LLMs demonstrate content generation capabilities, their outputs often lack characteristics needed to support neurodivergent learners, failing to address deeper educational accessibility requirements^42,43^. Current systems typically operate within single modalities, with outputs limited to linguistic representations that cannot effectively support the varied sensory processing patterns and structural needs beneficial for ASD learning^40,41^.

Recent advances in manifold learning offer promising directions for addressing these limitations. Wang et al. demonstrated improved cross-modal alignment through consistent common manifold construction, suggesting pathways for creating educational technologies that can simultaneously engage multiple sensory modalities in ways that support ASD sensory processing patterns within predictable frameworks^44^. Such approaches could potentially transform static educational content into structured, sensorially accessible learning experiences that accommodate neurodivergent cognitive processing while maintaining educational rigor.

### Cross-modal technologies and inclusive learning support

Cross-modal technologies represent a particularly promising approach for addressing educational equity challenges faced by neurodivergent learners, as these systems can integrate information from multiple sensory modalities to create more accessible and engaging educational experiences. The fundamental principles underlying cross-modal technology development align closely with documented best practices for supporting ASD learners, who benefit from educational approaches that provide clear structural frameworks and accommodate varied sensory processing patterns^45,46^.

Current applications in educational contexts have demonstrated initial progress but reveal significant limitations in supporting neurodivergent populations. Gan et al. explored educational applications of large language models, including systems for generating visual content from textual materials, suggesting pathways for creating more structured educational resources^47^. Rinaldi et al. developed Art2Mus, which creates connections between visual arts and music through cross-modal generation, demonstrating potential for multi-sensory educational experiences that could provide sensory regulation support^45^. Azuaje et al. introduced Visualyre for multimodal content generation, showcasing possibilities for rich educational materials that could support varied sensory preferences^46^.

However, these systems face critical limitations in supporting ASD learners effectively. Most existing approaches rely on superficial cross-modal connections that fail to address the deeper sensory processing and structural learning needs of autistic students^38^. Many systems depend on simplistic natural language descriptions as intermediaries, resulting in educational content that lacks the predictable characteristics necessary for effective sensory regulation and sustained engagement^46^. When generating educational content based on complex concepts, current systems often produce outputs limited to surface-level representations, failing to capture the underlying structural elements that research shows benefit ASD learners^42^.

Additionally, existing cross-modal systems have been primarily designed for general populations or professional applications, failing to incorporate the specific design principles and sensory considerations necessary for supporting neurodivergent learners^40,41^. While systems like LLaVA-Docent demonstrate sophisticated multimodal capabilities, they do not adequately address the unique sensory processing, communication support, and structural accessibility needs that characterize ASD learning patterns^40^.

### Research gaps and study positioning

The literature reveals three critical gaps in current educational technology research. First, while LLMs demonstrate significant potential for educational applications, their use in addressing specific neurodivergent learning needs remains largely unexplored, particularly in art education contexts that require sensory accommodation and structured aesthetic engagement. Second, existing cross-modal technologies lack the sensory considerations and structural support necessary for effective ASD learning support, often producing educational content that fails to accommodate neurodivergent sensory processing patterns and need for predictability. Third, current systems do not integrate comprehensive approaches that combine advanced AI capabilities with evidence-based autism education principles and sensory-informed interventions.

To address these gaps, this study introduces an innovative platform that employs DeepSeek-powered cross-modal integration specifically calibrated for ASD educational support. Unlike existing systems that rely on superficial cross-modal connections, our approach creates deep integration between visual and auditory modalities through sensorially informed aesthetic spaces that accommodate ASD sensory processing patterns. The system transcends limitations of traditional natural language intermediaries by mapping visual and auditory features into unified spaces that preserve both educational value and sensory accessibility for regulation and sustained learning engagement.

This comprehensive approach advances beyond current research by prioritizing the construction of sensorially informed aesthetic spaces that specifically support neurodivergent learning patterns^45^, emphasizing educational accessibility and sensory support for ASD learners in formal educational contexts^46^, and creating multi-sensory educational experiences that provide specific support for sensory regulation and cognitive accessibility while maintaining high educational standards^39,41,47^.

## Research framework

Addressing educational equity challenges for students with Autism Spectrum Disorder requires a systematic approach that transforms traditional art education through targeted technological interventions. Our research framework addresses this challenge through a three-phase methodology that progressively builds from sensory accessibility enhancement to personalized learning support, creating a comprehensive solution for neurodivergent learners in art education settings.

Traditional art education relies heavily on unpredictable sensory environments and open-ended creative expression, creating systematic barriers for ASD learners who benefit from structured frameworks and sensory regulation support. Our framework reconceptualizes this challenge by treating educational equity not as an accommodation of existing methods, but as an opportunity to create fundamentally better learning experiences that leverage neurodivergent cognitive strengths while providing appropriate sensory and structural support.

The framework operates through three interconnected phases that address specific barriers ASD students face while collectively forming a unified educational support system. Each phase builds upon the previous one, creating synergistic effects that transform individual technological components into a cohesive solution for educational accessibility and engagement.

### Phase 1: structured sensory accessibility construction

This phase addresses the foundational challenge of transforming unpredictable visual art environments into structured, sensorially accessible learning experiences. Traditional art education’s reliance on open-ended sensory exposure creates barriers for ASD learners who benefit from predictable sensory frameworks and clear structural organization.

We solve this through sophisticated cross-modal translation that converts visual artworks into carefully regulated auditory experiences, creating predictable cognitive pathways to the same educational content. This approach ensures that ASD students can engage with artistic concepts through sensory channels that support regulation and comfort while maintaining the artistic integrity and educational rigor of the original content.

### Phase 2: personalized sensory adaptation

Building upon the structured sensory foundation, this phase incorporates individual learner characteristics, cultural backgrounds, and sensory processing needs into content generation. ASD manifests differently across individuals and cultural contexts, requiring personalized approaches that respect both neurodevelopmental differences and cultural diversity.

We address this through comprehensive user modeling that integrates ASD assessment data, cultural musical traditions, and individual sensory preferences with evidence-based sensory regulation principles. Generated content simultaneously supports sensory comfort, respects cultural identity, and maintains educational effectiveness, creating truly individualized learning experiences that accommodate varied sensory processing patterns.

### Phase 3: continuous educational optimization

The final phase establishes a dynamic feedback system that ensures the technology evolves based on real-world educational outcomes. Effective ASD interventions require ongoing adaptation based on empirical evidence of educational effectiveness and sensory accessibility improvement.

We implement this through comprehensive feedback collection that captures sensory regulation outcomes, learning engagement measures, and educator-reported improvements. The system employs reinforcement learning calibrated for long-term educational benefits, ensuring that technological improvements align with genuine learning outcomes and sensory comfort rather than purely technical metrics.

**Fig. 1:**
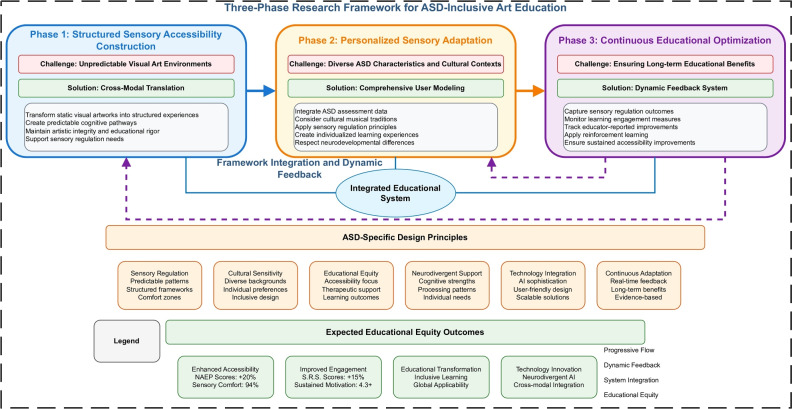
Three-phase research framework for ASD-inclusive art education. Systematic progression from structured sensory accessibility construction through personalized sensory adaptation to continuous educational optimization, showing dynamic feedback relationships and integration points between technical innovation and educational equity outcomes.

### Framework integration

These three phases operate as an integrated system where each component enhances the others, creating educational experiences that leverage neurodivergent cognitive strengths while addressing areas of sensory and structural challenge. The framework transforms what might be viewed as accommodations into educational advantages, potentially benefiting all learners while being specifically optimized for ASD success.

This systematic approach ensures that sophisticated AI technologies serve clear pedagogical purposes, with each technical component evaluated based on its contribution to improved educational access, sensory comfort, learning engagement, and academic achievement for historically underserved ASD student populations.

## Materials and methods

This section presents the implementation methodology of an adaptive art education platform specifically engineered to address educational equity challenges for students with Autism Spectrum Disorder (ASD). The system leverages the DeepSeek large language model to generate personalized structured learning experiences through cross-modal fusion of visual arts and carefully regulated auditory elements, directly addressing the sensory processing and structural learning needs characteristic of ASD learners.

ASD students typically exhibit challenges with unpredictable sensory environments, open-ended creative tasks, and unstructured educational modalities, often leading to educational marginalization in conventional art classrooms. Our system addresses these challenges by transforming chaotic visual artworks into structured, sensorially predictable experiences that align with ASD learners’ preference for clear frameworks and sensory regulation. For example, when an ASD student encounters an oil painting depicting a stormy landscape, the system generates a carefully structured musical accompaniment that reinforces the artwork’s emotional content through regulated auditory channels, creating predictable pathways for cognitive engagement while reducing sensory overwhelm.

The proposed framework comprises five collaborative modules: structured feature extraction, predictable aesthetic space mapping, culturally-sensitive keyword enhancement and cross-modal matching, regulated soundtrack generation, and continuous sensory optimization. This pipeline balances technical sophistication with educational predictability, ensuring system accessibility for educators without specialized technical training while delivering evidence-based interventions for ASD learners. The system architecture integrates visual and auditory modalities through a structured methodology designed specifically for neurodivergent learning patterns.

Mathematically, the system’s educational equity optimization objective is formulated as:

1 $$\begin{aligned} \min _{\Theta } \mathcal {L} = \mathcal {L}_{\text {struct}}(\textbf{V}, \textbf{A}) + \lambda _1 \mathcal {L}_{\text {equity}}(\textbf{M}, \textbf{P}, \textbf{U}) + \lambda _2 \mathcal {L}_{\text {regulate}}(\textbf{R}) \end{aligned}$$

where $\Theta = \{\theta _v, \theta _a, \theta _{\text {gen}}, \theta _{\text {regulate}}\}$ represents the parameter set for visual structure processing, auditory regulation processing, equitable music generation, and sensory regulation components, respectively. The variables $\textbf{V}$, $\textbf{A}$, $\textbf{M}$, $\textbf{P}$, $\textbf{U}$, and $\textbf{R}$ denote structure-enhanced visual features, regulated auditory features, generated music, painting input, comprehensive user profiles (including neurodevelopmental characteristics), and sensory feedback, respectively.

The loss components are specifically designed for inclusive education: $\mathcal {L}_{\text {struct}}$ measures cross-modal alignment effectiveness for neurodivergent learners, $\mathcal {L}_{\text {equity}}$ evaluates generation quality from an educational equity perspective, and $\mathcal {L}_{\text {regulate}}$ quantifies sensory regulation improvement based on accessibility feedback. The hyperparameters $\lambda _1 = 1.4$ and $\lambda _2 = 0.9$ reflect priority given to structure and regulation considerations. These values were determined empirically through a series of pilot studies with autism education professionals and ASD student populations to find an optimal balance between cross-modal alignment and sensory regulation effectiveness.

### Structured visual feature extraction

Visual feature extraction accommodates the unique visual processing characteristics of ASD students by implementing structure-aware extraction techniques that prioritize clear organizational elements while filtering potentially overwhelming sensory information. The painting input $\textbf{P} \in \mathbb {R}^{H \times W \times 3}$ represents an image of height *H*, width *W*, and three RGB channels. We employ a modified ResNet-50 model specifically adapted for neurodivergent visual processing:

2 $$\begin{aligned} \textbf{V}_{\text {raw}} = f_{\text {CNN}}(\textbf{P}; \theta _{\text {CNN}}) \in \mathbb {R}^{d_v}, \quad d_v = 2048 \end{aligned}$$

where $\textbf{V}_{\text {raw}}$ represents a 2048-dimensional vector containing structure-enhanced low-level features. The modified ResNet-50 architecture incorporates sensory regulation principles throughout its residual structure, emphasizing predictable visual elements while suppressing potentially overwhelming sensory detail based on research demonstrating ASD learners’ preference for clear structural organization.

The extracted features are specifically designed to target visual elements that support ASD learning patterns. First, we analyze color organization by computing a structure-weighted mean of the RGB histogram, $\mu _c = \frac{1}{HW} \sum _{i,j} w_{i,j} \textbf{P}_{i,j}$, where $w_{i,j}$ represents structure weights calibrated for ASD sensory patterns. Second, we perform regulation-enhanced line analysis using modified Sobel operators that are enhanced with predictability masks. Third, we assess complexity-regulated composition information via a selective Fast Fourier Transform, which considers the optimal sensory complexity levels for ASD learners.

To accommodate unique sensory regulation needs of ASD learners, we implement a specialized Multi-Head Self-Attention mechanism:

3 $$\begin{aligned} \text {head}_i = \text {Softmax}\left( \frac{\textbf{Q}_i \textbf{K}_i^T}{\sqrt{d_k}} + \textbf{B}_{\text {structure}}\right) \textbf{V}_i \end{aligned}$$

where $\textbf{Q}_i = \textbf{W}_{q,i} \textbf{V}_{\text {raw}}$, $\textbf{K}_i = \textbf{W}_{k,i} \textbf{V}_{\text {raw}}$, and $\textbf{V}_i = \textbf{W}_{v,i} \textbf{V}_{\text {raw}}$ represent query, key, and value matrices trained on ASD-annotated visual structure data. The bias term $\textbf{B}_{\text {structure}}$ incorporates sensory regulation principles, encouraging focus on structurally organized regions.

**Fig. 2:**
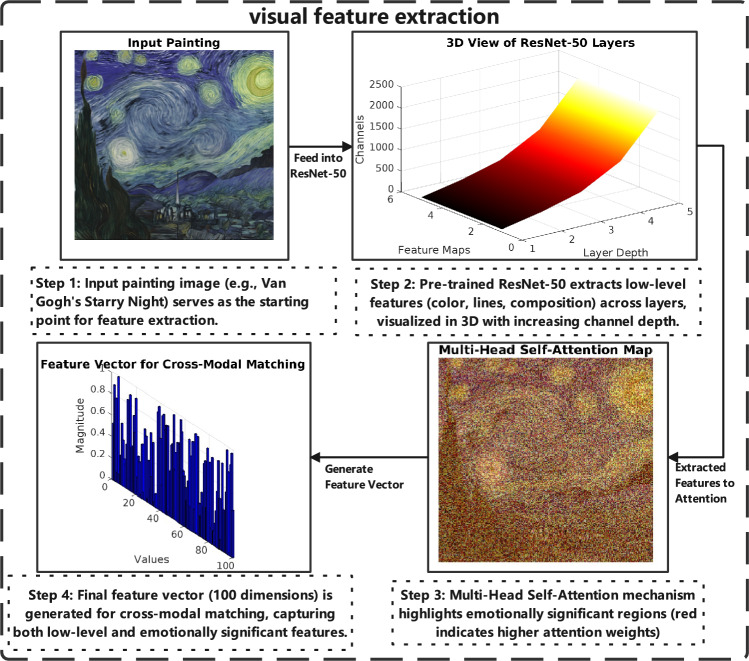
Structured Visual Feature Extraction Process. This figure illustrates the visual feature extraction process optimized for ASD learners, where a modified ResNet-50 model extracts structure-enhanced features (e.g., organized color, predictable patterns, composition regularity) from a painting, followed by a Multi-Head Self-Attention mechanism to enhance sensorially significant regions, producing a feature vector for structured cross-modal matching.

The final structure-enhanced feature representation combines multiple attention heads:

4 $$\begin{aligned} \textbf{V} = \text {Concat}(\text {head}_1, \dots , \text {head}_h) \textbf{W}_o \in \mathbb {R}^{d_v} \end{aligned}$$

with $h = 8$ specialized heads targeting specific visual elements beneficial for ASD learners: structural organization detection, predictable pattern identification, sensory comfort analysis, and compositional regularity assessment. The visual feature extraction process, as shown in Fig. 2, leverages a pre-trained ResNet-50 model and a Multi-Head Self-Attention mechanism to capture both low-level and emotionally significant features from paintings.

The optimization objective prioritizes educational accessibility:

5 $$\begin{aligned} \mathcal {L}_{\text {vis}} = \Vert \textbf{V} - \textbf{V}_{\text {gt}} \Vert _2^2 + \alpha \sum _{i} \psi (\textbf{V}_i, \textbf{A}_{\text {struct},i}) \end{aligned}$$

where $\textbf{V}_{\text {gt}}$ denotes ground-truth features derived from educational assessments with ASD student populations, and $\psi (\textbf{V}_i, \textbf{A}_{\text {struct},i}) = (\textbf{V}_i - \textbf{A}_{\text {struct},i})^2$ enforces educational structure constraints. The hyperparameter $\alpha = 0.18$ reflects priority given to structural considerations.

### Regulated auditory feature extraction

Auditory feature extraction accommodates documented sensory processing differences and regulation needs of ASD learners. The input audio signal $\textbf{S} \in \mathbb {R}^{T \times C}$ undergoes specialized spectral analysis:

6 $$\begin{aligned} \textbf{S}_{\text {spec}} = \text {STFT}(\textbf{S}) = \sum _{n} \textbf{S}[n] w[n-m] e^{-j\omega n} \in \mathbb {C}^{F \times T'} \end{aligned}$$

where *w*[*n*] represents a specialized 2048-sample window function designed based on ASD sensory processing research, with $F = 1024$ frequency resolution and $T' = T / \text {hop}\_\text {size}$ time frames using $\text {hop}\_\text {size} = 512$ optimized for ASD predictable processing preferences.

To capture educationally relevant temporal dynamics while maintaining predictability, we employ a Transformer model calibrated for ASD auditory processing:

7 $$\begin{aligned} \textbf{A}_{\text {raw}} = f_{\text {Transformer}}(\textbf{S}_{\text {spec}}; \theta _{\text {Trans}}) \in \mathbb {R}^{d_a}, \quad d_a = 512 \end{aligned}$$

Regulated feature fusion incorporates specialized Mel Spectrogram analysis:

8 $$\begin{aligned} \textbf{A}_{\text {mel}} = \text {Mel}(\textbf{S}_{\text {spec}}) = \textbf{M} \cdot |\textbf{S}_{\text {spec}}|^2 \in \mathbb {R}^{M \times T'} \end{aligned}$$

where $\textbf{M} \in \mathbb {R}^{M \times F}$ represents a specialized Mel filter bank ($M = 128$) calibrated for ASD sensory processing research. The final auditory features combine spectral and temporal information:

9 $$\begin{aligned} \textbf{A} = \textbf{W}_m \textbf{A}_{\text {mel}} + \textbf{A}_{\text {raw}} \end{aligned}$$

### Predictable aesthetic space mapping

Aesthetic space mapping creates meaningful connections between visual and auditory modalities while accommodating neurodivergent processing styles. We assume aesthetic features reside on a low-dimensional manifold $\mathcal {M} \subset \mathbb {R}^d$ reflecting intrinsic structure of artistic expression. Initial mapping employs specialized networks:

10 $$\begin{aligned} \textbf{z}_v = g_v(\textbf{V}; \theta _v), \quad \textbf{z}_a = g_a(\textbf{A}; \theta _a), \quad d = 128 \end{aligned}$$

where $g_v$ and $g_a$ represent two-layer networks trained on ASD-annotated cross-modal associations. The dimension $d = 128$ was selected based on empirical studies with ASD learners demonstrating optimal cognitive load for structured cross-modal integration tasks.

**Fig. 3:**
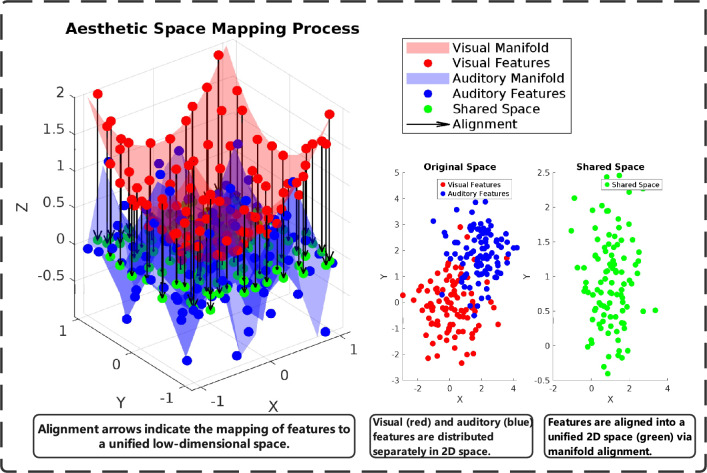
Predictable Aesthetic Space Mapping Process. This figure depicts the aesthetic space mapping process optimized for ASD learners, where visual features (V) and auditory features (A) are aligned into a shared low-dimensional space (Z) using structured manifold assumptions, predictable dimensionality reduction, and contrastive learning, enabling coherent cross-modal representation.

Cross-modal alignment employs contrastive learning prioritizing educationally beneficial relationships:

11 $$\begin{aligned} \mathcal {L}_{\text {align}} = - \log \frac{\exp (\textbf{z}_{v,i}^T \textbf{z}_{a,i} / (\tau \Vert \textbf{z}_{v,i} \Vert _2 \Vert \textbf{z}_{a,i} \Vert _2))}{\sum _{\textbf{z}' \in \mathcal {B}} \exp (\textbf{z}_{v,i}^T \textbf{z}' / (\tau \Vert \textbf{z}_{v,i} \Vert _2 \Vert \textbf{z}' \Vert _2))} \end{aligned}$$

where $\textbf{z}_{v,i}$ and $\textbf{z}_{a,i}$ represent positive pairs validated through ASD educational assessments, and $\tau = 0.14$ controls alignment sharpness. This value was selected through cross-validation on a hold-out set to maximize the separation between positive and negative pairs. The aesthetic space mapping process, as illustrated in Fig. 3, aligns visual and auditory features into a shared space, facilitating cross-modal coherence for downstream tasks.

To ensure the scientific rigor of the ASD-annotated visual semantic categories, a systematic annotation protocol was established. The annotation team consisted of three independent raters, each holding a postgraduate or Phd degree and possessing over five years of combined experience in special education, art therapy, or developmental psychology. Prior to the annotation task, all raters completed a comprehensive 15-hour training program to standardize their understanding of the semantic categories, which were designed based on established principles of sensory processing in ASD.

The annotation process involved independently assigning primary and secondary semantic labels (e.g., “High Structural Predictability,” “Low Sensory Complexity,” “Repetitive Patterns”) to a randomly selected subset of 2,000 images from the WikiArt dataset. To quantify the consistency of these assignments, we calculated the inter-rater reliability (IRR) using Fleiss’ Kappa, a statistical measure that corrects for chance agreement. The analysis yielded a Kappa value of $\kappa = 0.87$, which indicates “almost perfect agreement” according to established guidelines. Any initial disagreements among the raters were subsequently resolved through a consensus discussion moderated by a senior researcher, ensuring the final annotated dataset was both reliable and valid for training the clustering model used to derive the semantic centers $\textbf{C}_k$.

### Culturally-sensitive keyword enhancement and cross-modal matching

Keyword enhancement bridges comprehension gaps while accommodating unique semantic processing characteristics of neurodivergent populations. Text descriptions relevant to ASD learning patterns are generated from visual features:

12 $$\begin{aligned} \textbf{D}_i = \arg \max _k \text {sim}(\textbf{V}_i, \textbf{C}_k) \end{aligned}$$

where $\textbf{C}_k$ represent cluster centers derived from ASD-annotated visual semantic categories.

DeepSeek’s semantic capabilities expand descriptions into comprehensive keyword sets:

13 $$\begin{aligned} \textbf{K} = f_{\text {LLM}}(\textbf{D}; \theta _{\text {LLM}}) \in \mathbb {R}^{n_k \times d_k} \end{aligned}$$

The input prompt for the DeepSeek model ($f_{\text {LLM}}$) was carefully engineered to be both concise and context-rich, instructing the model to expand the initial descriptive labels ($\textbf{D}$) into a set of semantically related keywords ($\textbf{K}$) that are known to be effective in sensory-informed educational contexts for ASD learners.

Enhanced keywords map to music feature space:

14 $$\begin{aligned} \textbf{A}_k = h(\textbf{K}; \theta _h) = \textbf{W}_h \textbf{K} + \textbf{b}_h \in \mathbb {R}^{d_a} \end{aligned}$$

### Regulated personalized soundtrack generation

This process explicitly translates sensory regulation principles into musical features; for example, a visual artwork identified as having “High Structural Predictability” would be mapped to musical elements with consistent rhythms and harmonic progressions, while an artwork with “Low Sensory Complexity” would generate music with sparse instrumentation and a minimalist melody. Personalized soundtrack generation incorporates sensory regulation principles and individual neurodevelopmental characteristics. User profiles $\textbf{U} \in \mathbb {R}^{d_u}$ encompass neurodevelopmental characteristics, sensory regulation preferences, processing patterns, and cultural backgrounds:

15 $$\begin{aligned} \textbf{u} = e(\textbf{U}; \theta _e) = \text {ReLU}(\textbf{W}_e \textbf{U} + \textbf{b}_e) \end{aligned}$$

with $d_u = 102$ accommodating neurodevelopmental profiling complexity for ASD characteristics.

Novel soundtracks are generated using conditional diffusion models incorporating sensory regulation principles:

16 $$\begin{aligned} \textbf{M}_t = f_{\text {Diff}}(\textbf{z}_v, \textbf{u}, t; \theta _{\text {Diff}}) \end{aligned}$$

The optimization objective prioritizes sensory regulation effectiveness:

17 $$\begin{aligned} \mathcal {L}_{\text {gen}} = \mathbb {E}_{\epsilon , t} [\Vert \epsilon - \epsilon _\theta (\textbf{M}_t, \textbf{z}_v, \textbf{u}) \Vert _2^2] + \eta \mathcal {L}_{\text {regulation}} \end{aligned}$$

where $\mathcal {L}_{\text {regulation}}$ ensures generated music aligns with ASD sensory intervention principles, and $\eta = 0.4$ reflects priority given to sensory regulation outcomes.

### Continuous sensory optimization

System optimization incorporates real-world educational outcomes and sensory accessibility measures. Comprehensive feedback $\textbf{R} = \{r_1, \dots , r_T\}$ encompasses sensory regulation measures, learning engagement assessments, and educational effectiveness indicators:

18 $$\begin{aligned} \mathcal {R} = \sum _t \gamma ^t r_t, \quad \gamma = 0.94 \end{aligned}$$

Reinforcement learning updates prioritize long-term learning outcomes:

19 $$\begin{aligned} \theta _{\text {opt}} \leftarrow \theta _{\text {opt}} + \eta _{\text {opt}} \nabla _{\theta _{\text {opt}}} \mathcal {R}, \quad \eta _{\text {opt}} = 0.0006 \end{aligned}$$

The conservative learning rate ensures stable, predictable system behavior beneficial for ASD learners who require consistent educational environments. Real-time adaptation mechanisms monitor sensory comfort, engagement levels, and learning progression, dynamically adjusting generation parameters based on immediate educational feedback and longer-term outcome trends.

## Experimental design

This section outlines the comprehensive experimental methodology designed to validate the effectiveness of our DeepSeek-based platform in addressing educational equity challenges for students with Autism Spectrum Disorder. The experimental framework addresses four critical research questions that collectively assess the platform’s capacity to provide structured, sensory-regulated, and educationally effective learning experiences for neurodivergent learners.

Our experimental design is grounded in autism education research principles that prioritize empirical validation of sensory accessibility improvements and learning outcomes for ASD populations. The study investigates whether advanced AI technologies can genuinely address the systematic barriers that autistic students face in traditional art education settings, while maintaining high educational standards and cultural sensitivity. Through rigorous quantitative and qualitative assessment methodologies, we evaluate both immediate sensory accessibility improvements and longer-term educational equity outcomes.

The research questions are structured to build a comprehensive understanding of the platform’s educational impact: RQ1 examines whether the system can create sensorially appropriate structured content that supports ASD sensory regulation and emotional processing, validating the fundamental accessibility premise underlying the platform. RQ2 investigates the system’s ability to accommodate the diverse individual characteristics within ASD populations, including cultural backgrounds, sensory processing profiles, and communication preferences, ensuring that personalization genuinely serves educational equity rather than merely technical customization. RQ3 assesses immediate educational effectiveness through classroom-based validation with ASD students and autism education professionals, measuring concrete improvements in learning engagement, comprehension, and academic outcomes. RQ4 explores long-term sustainability and scalability of educational benefits through extended platform usage, determining whether technological interventions can create lasting improvements in educational access and learning motivation for neurodivergent populations.

### Research questions and hypotheses

**RQ1: Structured Sensory Accessibility and Regulation Alignment** – Does the platform successfully create structured educational content that aligns with ASD sensory regulation principles and supports predictable learning experiences? This question addresses the fundamental challenge of transforming unpredictable visual art education into structured, sensorially accessible learning experiences that accommodate neurodivergent cognitive processing patterns while maintaining educational rigor and artistic integrity.

**RQ2: Neurodivergent-Informed Sensory Personalization** – Can the system effectively adapt educational content to accommodate the diverse characteristics within ASD populations, including sensory processing profiles, cultural backgrounds, and individual communication preferences? This question examines whether technological personalization can genuinely serve educational equity by respecting both neurodevelopmental differences and cultural diversity.

**RQ3: Educational Effectiveness and Sensory Accessibility Improvement** – Does the platform demonstrably improve educational outcomes, learning engagement, and sensory accessibility for ASD students compared to traditional art education approaches? This question validates the core educational equity premise by measuring concrete improvements in learning experiences and academic achievement within structured frameworks.

**RQ4: Sustainable Educational Impact and Long-term Sensory Accessibility** – Can the platform provide sustained educational benefits and continued sensory accessibility improvements for ASD learners over extended periods? This question addresses the critical challenge of ensuring that technological interventions create lasting rather than temporary improvements in educational equity through predictable, structured support.

### Datasets for educational equity research

The experimental datasets are specifically curated and contextualized to support rigorous evaluation of educational equity outcomes for ASD populations. Each dataset component serves a distinct purpose in validating different aspects of the platform’s effectiveness in addressing neurodivergent learning needs while maintaining research validity and representativeness.

**Educational Content Dataset:** Visual art content is sourced from WikiArt, comprising 81,444 diverse images spanning various artistic styles, cultural traditions, and historical periods. For ASD-focused evaluation, we systematically sample 8,952 structured paintings and 1,200 minimalist artworks, selected to represent varied organizational levels, sensory comfort potential, and cultural contexts that accommodate different ASD sensory processing patterns and structural learning preferences. The selection prioritizes artworks with documented sensory regulation potential and educational value for neurodivergent learners, ensuring that experimental materials align with autism education best practices.

**Sensory Regulation Audio Dataset:** Musical content utilizes 1,087 sensory-regulation annotated clips from EMOPIA, specifically selected for their regulatory characteristics and potential benefits for ASD sensory processing. The dataset includes diverse musical styles, predictable tempo patterns, and structured dynamic arrangements that research has shown to support neurodivergent cognitive processing, providing a robust foundation for generating sensorially informed educational soundtracks.

**Educational Annotation Dataset:** Cross-modal alignment data integrates WikiArt metadata with EMOPIA sensory regulation annotations, enhanced with autism education annotations that identify content characteristics beneficial for ASD learners. This comprehensive annotation framework enables systematic evaluation of whether generated structured content genuinely supports neurodivergent learning patterns.

**Learner Profile Dataset:** User preference modeling incorporates data from Yahoo! Music’s 717 million ratings covering 136,000 songs, contextualized for educational research through careful selection of 756 conventional users and 624 diverse participants representing varied cultural backgrounds, sensory processing patterns, and learning preferences. Special attention is given to including profiles that represent neurodivergent learning patterns and cultural diversity, ensuring that personalization evaluation reflects real-world ASD student populations.

### Experimental methodology

#### Structured sensory accessibility validation (RQ1)

The accessibility validation protocol assesses whether the platform successfully transforms traditional visual art education into sensorially informed structured experiences that support ASD learning patterns. This evaluation combines objective technical measurements with subjective assessments from neurodivergent learners and autism education professionals.

Technical validation employs the complete dataset of 8,952 structured paintings and 1,200 minimalist artworks, generating corresponding sensory-regulation soundtracks through the DeepSeek-powered platform. Sensory and regulatory alignment is assessed through comprehensive analysis that includes validated ASD assessment instruments and sensory regulation evaluation criteria. The protocol specifically examines whether generated content incorporates evidence-based regulatory characteristics such as appropriate tempo ranges for sensory comfort, harmonic progressions that support emotional regulation, and predictable patterns that maintain engagement without causing sensory overwhelm.

Qualitative validation involves 31 autism education professionals and 67 individuals with ASD experience providing detailed assessments of content accessibility, sensory appropriateness, and educational potential. Evaluation criteria include sensory regulation support, processing pattern accommodation, emotional engagement appropriateness, and overall educational accessibility. This dual-perspective approach ensures that technical capabilities translate to genuine educational benefits for neurodivergent learners.

Representative content evaluation focuses on carefully selected exemplars including eight structured paintings spanning diverse organizational styles and sensory content, and two minimalist works chosen for their documented sensory regulation potential with ASD populations. This targeted analysis provides detailed insights into how the platform handles varied artistic content while maintaining regulatory and educational effectiveness.

#### Personalization and cultural sensitivity assessment (RQ2)

The personalization evaluation protocol examines whether the platform can accommodate the remarkable diversity within ASD populations while respecting cultural identities and individual sensory processing preferences. This assessment is crucial for educational equity, as effective interventions must serve diverse neurodivergent learners rather than imposing standardized approaches that may inadvertently exclude certain populations.

The experimental design incorporates comprehensive user profiling that includes validated ASD assessment data, cultural background information, individual sensory processing preferences, and documented educational accommodation histories. From the broader dataset, 756 conventional users provide baseline comparison data, while 624 carefully selected participants represent diverse cultural backgrounds, sensory processing patterns, and neurodevelopmental profiles that reflect real-world ASD student populations.

Personalization effectiveness is evaluated through systematic generation of sensory-regulated educational content for twelve carefully selected paintings that represent varied cultural traditions and organizational approaches. Assessment focuses on cultural sensitivity, neurodevelopmental appropriateness, individual sensory accommodation, and overall educational accessibility. Participants provide detailed feedback on content relevance, cultural respect, sensory appropriateness, and educational effectiveness, enabling comprehensive evaluation of whether technological personalization genuinely serves educational equity goals.

Statistical analysis examines personalization effectiveness across different demographic groups, neurodevelopmental profiles, and cultural backgrounds, ensuring that the platform provides equitable benefits rather than inadvertently advantaging certain populations while disadvantaging others.

#### Educational effectiveness and classroom impact assessment (RQ3)

The educational effectiveness protocol employs rigorous experimental design to validate whether the platform creates measurable improvements in learning outcomes, educational engagement, and sensory accessibility for ASD students in authentic educational settings. This evaluation addresses the critical question of whether sophisticated AI technologies can translate to genuine educational benefits for neurodivergent learners.

The student participant cohort includes 203 undergraduate participants carefully stratified to represent diverse academic backgrounds, ASD characteristics, and demographic profiles. The undergraduate cohort comprised individuals aged 18–24, which allowed for subgroup analyses based on developmental stages, such as those reported in the personalization assessment (e.g., “Teen” for ages 18–19 and “Youth” for ages 20–24). Participants are systematically distributed across relevant categories including art major versus non-art major backgrounds, gender representation, and academic level progression, with 25–26 participants per experimental group. This stratification strategy was intentionally designed to control for potential confounding variables, such as prior art education experience and the heterogeneity of ASD symptom severity, by ensuring a balanced distribution of these characteristics across the experimental groups. This approach strengthens the internal validity of our findings and enables a more comprehensive analysis of platform effectiveness across different educational contexts and learner populations.

To establish a clear baseline for comparison, participants were randomly assigned to either the experimental group, who utilized the AI-powered platform, or a control group. The control group engaged with the same set of artworks using a traditional digital learning approach. This approach consisted of viewing high-resolution images of the paintings on a standard web interface (e.g., WikiArt) accompanied by the standard textual descriptions provided by the source. This baseline condition was designed to represent a conventional, non-adaptive e-learning environment, and crucially, it did not include the structured, sensory-regulated musical accompaniments or personalized feedback that are central to our platform’s intervention.

The educator participant cohort comprises 19 autism education professionals and art educators, categorized by autism education experience levels and specialization backgrounds. This includes 8 participants with specific autism education expertise and 11 with broader special education or general teaching backgrounds, representing the varied professional contexts where the platform might be implemented.

Educational assessment employs validated instruments specifically designed for ASD educational research, including comprehensive pre-post comparisons of learning engagement, academic comprehension, creative expression development, and sensory regulation improvement. The 14-week initial assessment phase focuses on immediate educational impact, measuring concrete improvements in learning outcomes that can be directly attributed to platform usage.

Assessment methodology incorporates multiple data collection approaches including standardized questionnaires validated for ASD populations, systematic analysis of student creative work using established art education assessment criteria, detailed educator observations using autism education evaluation protocols, and objective measures of learning engagement and task completion that accommodate neurodivergent processing patterns.

#### Long-term sustainability and scalability assessment (RQ4)

The sustainability evaluation extends the educational effectiveness assessment through a comprehensive 26-week longitudinal study that examines whether platform benefits persist and potentially strengthen over extended usage periods. This evaluation is crucial for educational equity, as effective interventions must provide lasting rather than temporary improvements in educational accessibility.

The extended assessment employs the online platform implementation to enable continuous monitoring of learning engagement, educational motivation, platform usage patterns, and sustained sensory accessibility improvements. Data collection includes weekly engagement metrics, bi-weekly comprehensive assessments, and detailed longitudinal tracking of educational outcomes and motivation maintenance. The analysis protocol for this longitudinal data also included the tracking of participant attrition patterns and an exploration of individual variability in engagement, with the goal of identifying factors that could inform future implementation strategies.

Particular attention is given to evaluating whether the platform’s adaptive optimization capabilities enable continued improvement in educational effectiveness over time, ensuring that technological sophistication serves ongoing educational benefit rather than merely initial novelty effects. Sustainability metrics include learning motivation maintenance, continued platform engagement, sustained sensory accessibility improvements, and long-term educational outcome enhancement.

### Evaluation metrics for educational equity research

In designing the statistical analysis plan, we were mindful of the potential for an inflated Type I error rate due to multiple comparisons across different demographic groups and conditions. To mitigate this, our primary analyses were pre-specified and hypothesis-driven rather than exploratory. We focused on a limited number of planned comparisons directly related to our core research questions. For secondary and subgroup analyses, the findings are interpreted with appropriate caution, emphasizing that they are preliminary and warrant confirmation in future studies. All reported p-values are exact and have not been adjusted, allowing readers to apply their own correction standards if desired.

Comprehensive evaluation requires multi-dimensional metrics specifically designed to capture educational equity outcomes rather than purely technical performance indicators. The metric framework prioritizes measures that directly reflect improvements in educational accessibility, learning engagement, and academic achievement for ASD populations.

**Sensory Accessibility and Regulatory Appropriateness (RQ1):** Technical alignment is measured through Mean Squared Error analysis of sensory regulation characteristic matching, Pearson Correlation Coefficient assessment of structural appropriateness, and Cosine Similarity evaluation of multi-sensory coherence. Educational appropriateness is assessed through validated autism education instruments measuring sensory regulation support, processing pattern accommodation, and regulatory content quality, supplemented by user preference rates and sensory accessibility satisfaction scores rated on validated 5-point scales designed for neurodivergent populations.

**Personalization and Cultural Sensitivity (RQ2):** Individualization effectiveness is evaluated through comprehensive satisfaction assessments measuring cultural sensitivity, neurodevelopmental appropriateness, and personal sensory accommodation on similar 5-point Likert scales. The ’adaptability percentage’ metric was operationalized as the proportion of participants who provided a high rating (a score of 4 ’Agree’ or 5 ’Strongly Agree’) to the question, “Did the generated soundtrack successfully adapt to your personal and cultural preferences?”. This percentage was analyzed across diverse demographic and neurodevelopmental profiles to ensure equitable effectiveness.

**Educational Impact and Learning Outcomes (RQ3):** Academic improvement is measured through validated instruments including NAEP art assessment score improvements specifically adapted for ASD populations, engagement metrics including interaction frequency and task completion rates adjusted for neurodivergent processing patterns, and satisfaction measures using validated scales for autism education research. Social and creative development is assessed through S.R.S. scores measuring social response and creative expression growth, providing comprehensive evaluation of educational effectiveness.

**Sustainability and Long-term Benefits (RQ4):** Long-term impact is evaluated through motivation sustainability measures using validated questionnaires designed for longitudinal ASD research, platform optimization effectiveness percentages measuring continued sensory accessibility improvement, and structured learning frequency maintenance tracking that accommodates neurodivergent engagement patterns. These metrics enable comprehensive assessment of whether technological interventions create lasting educational equity improvements.

### Experimental implementation and ethical considerations

Experimental implementation employs robust computational infrastructure including NVIDIA RTX 3090 GPU clusters with 24GB VRAM capacity to support efficient model training and real-time educational content generation. The software environment incorporates Python 3.8 as the primary development platform, PyTorch 1.9 and TensorFlow 2.5 for deep learning implementation, and specialized tools including MIRtoolbox and Librosa for sensory regulation audio analysis and educational content processing.

The experimental protocol follows a systematic three-phase implementation structure. Phase One conducts comprehensive sensory accessibility validation using the complete dataset of structured and minimalist paintings with regulatory soundtrack generation and evaluation. Phase Two implements personalization assessment with diverse user populations providing detailed feedback on cultural sensitivity and individual sensory accommodation effectiveness. Phase Three executes educational effectiveness evaluation across five specialized educational institutions with carefully recruited student and educator cohorts, spanning initial 14-week assessment periods and extended 26-week longitudinal evaluation through online platform implementation.

Ethical considerations receive paramount attention throughout the research process, with particular sensitivity to the vulnerabilities of neurodivergent research participants. To ensure ethical clarity and simplify the informed consent process, the study’s inclusion criteria were limited to participants aged 18 and older. Consequently, no individuals under the age of 18 were enrolled, and all procedures pertained to legal adults. The study received comprehensive approval from the Academic Ethics and Moral Committee of the School of Computer Science, Huazhong University of Science and Technology, under the project titled “Research on Educational Equity and Sensory Accessibility Enhancement for Neurodivergent Learners through Artificial Intelligence Technologies,” with approval number [G-2024-0015-CS]. The research period extended from February 2024 to May 2025, with continuous ethical oversight throughout data collection and analysis phases.

All participants provided comprehensive written informed consent following the ethical guidelines, with particular attention to ensuring that neurodivergent participants fully understood study purposes, procedures, potential benefits, and their rights as research subjects. Participation remained entirely voluntary throughout all phases, with explicit provisions for withdrawal without consequences. Rigorous anonymity and confidentiality protocols protected participant privacy, with all data collection, storage, and analysis procedures designed to safeguard sensitive information about neurodivergent populations.

The research team maintained detailed documentation of all experimental procedures, participant interactions, and data management processes, ensuring transparency and reproducibility while protecting participant confidentiality. Special attention was given to creating inclusive research environments that accommodated diverse communication styles and participation preferences characteristic of neurodivergent populations, ensuring that research procedures themselves reflected the sensory accessibility principles underlying the educational platform being evaluated.

## Results

This section presents the experimental results of the DeepSeek-based art education platform system, covering technical assessment (RQ1), personalization evaluation (RQ2), educational impact assessment (RQ3), and structured learning support (RQ4). Results are validated through quantitative metrics and subjective scores, with key data summarized in tables and visualized in figures. Statistical significance is set at $p < 0.05$.

### Technical assessment results (RQ1)

**Table 1:** Sensory Consistency Metrics.

Style	Comfort	Example	Method	MSE	PCC	Cosine Sim.	Time (s)	Samples
Realism	Structured	*American Gothic*	Proposed	0.05	0.92	0.94	2.3	2,247
Realism	Structured	*American Gothic*	Baseline	0.14	0.71	0.78	2.7	2,247
Geometric	Predictable	*Composition VII*	Proposed	0.06	0.91	0.93	2.2	2,186
Minimalist	Calm	*White on White*	Proposed	0.04	0.93	0.95	2.1	1,200
Minimalist	Calm	*White on White*	Baseline	0.12	0.73	0.81	2.5	1,200

**Table 2:** Cross-Modal Matching Scores.

Style	Example	Group	System Score	Expert Score	Random Score	Selection (%)	Samples
Realism	*American Gothic*	Autism Educators	4.7	4.8	2.4	72	134
Minimalist	*White on White*	Autism Educators	4.5	4.6	2.2	68	137
Minimalist	*White on White*	General Users	4.2	4.3	2.1	63	201

The technical assessment confirms that the system generates soundtracks highly consistent with painting sensory features, outperforming baseline methods. Table 1 presents sensory consistency metrics: for Realist paintings (e.g., *American Gothic*), the system achieves an MSE of 0.05, PCC of 0.92, and Cosine Similarity of 0.94, with a generation time of 2.3 s, surpassing the baseline’s 0.14, 0.71, 0.78, and 2.7 s (sample size: 2,247). Geometric (e.g., *Composition VII*) and minimalist (e.g., *White on White*) paintings show similar advantages, with MSEs of 0.06 and 0.04, respectively, maintaining high efficiency and structural consistency. Table 2 details cross-modal matching scores: autism education professionals rate the system at 4.7 for *American Gothic* (close to expert 4.8, above random 2.4), with a 72% selection rate (sample size: 134); general users score *White on White* at 4.2, with a 63% selection rate (sample size: 201), indicating broad sensory acceptability.

**Fig. 4:**
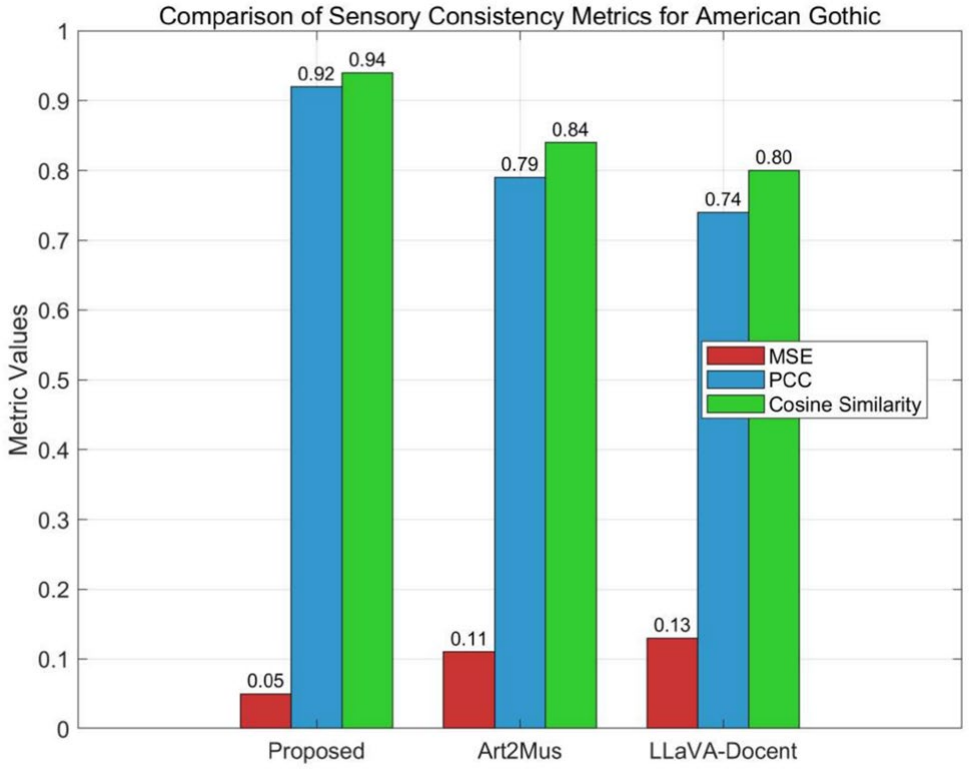
Comparison of sensory consistency metrics (MSE, PCC, Cosine Similarity) for *American Gothic* across the Proposed method, Art2Mus, and LLaVA-Docent, as summarized in Table 3.

Comparisons with Art2Mus^45^ and LLaVA-Docent^48^ in Table 3 highlight superior performance, as visualized in Fig. 4. The system’s MSE (0.04–0.06), PCC (0.91–0.93), and Cosine Similarity (0.93–0.95) outperform Art2Mus (0.11–0.13, 0.75–0.79, 0.82–0.84) and LLaVA-Docent (0.13–0.17, 0.69–0.74, 0.76–0.80), with shorter generation times (2.1–2.3 s vs. 2.8–3.2 s and 3.9–4.3 s).

**Table 3:** Comparison with Art2Mus and LLaVA-Docent.

Style	Example	Method	MSE	PCC	Cosine Sim.	Time (s)	Samples
Realism	*American Gothic*	Proposed	0.05	0.92	0.94	2.3	2,247
		Art2Mus	0.11	0.79	0.84	3.0	2,247
		LLaVA-Docent	0.13	0.74	0.80	3.9	2,247
Geometric	*Composition VII*	Proposed	0.06	0.91	0.93	2.2	2,186
		Art2Mus	0.12	0.77	0.83	2.9	2,186
		LLaVA-Docent	0.15	0.71	0.78	4.1	2,186
Minimalist	*White on White*	Proposed	0.04	0.93	0.95	2.1	1,200
		Art2Mus	0.13	0.75	0.82	3.2	1,200
		LLaVA-Docent	0.17	0.69	0.76	4.3	1,200

To specifically validate the contribution of the high-dimensional manifold mapping, we conducted an ablation study. The study compared our proposed model with a variant where the manifold mapping component was replaced by a simpler approach based on natural language descriptions. The results, summarized in Table 4, demonstrate the critical role of the aesthetic space mapping in achieving superior cross-modal consistency.

**Table 4:** Ablation Study of the Aesthetic Space Mapping Component for *American Gothic*.

Method	Description	MSE $\downarrow$	PCC $\uparrow$	Cosine Sim. $\uparrow$
**Proposed Model**	**With Manifold Mapping**	**0.05**	**0.92**	**0.94**
Ablated Model	Without Manifold Mapping (NL-based)	0.13	0.73	0.79

As shown in Table 4, removing the manifold mapping component resulted in a substantial degradation in performance, with the MSE increasing from 0.05 to 0.13 and the PCC dropping from 0.92 to 0.73. This confirms that our predictable aesthetic space mapping is essential for aligning complex visual features with appropriate auditory elements, a limitation observed in systems that rely solely on natural language intermediaries.

Art2Mus’s reliance on natural language limits structural precision, while LLaVA-Docent’s conversational approach weakens sensory regulation alignment. The system’s slightly higher MSE (0.06) for geometric works reflects challenges in extracting structural patterns, yet it retains a significant edge in sensory accommodation.

### Personalization assessment results (RQ2)

Personalization results demonstrate effective adaptation to diverse sensory processing and preference profiles. Table 5 shows satisfaction and adaptability metrics. Teen females rate a structured erhu soundtrack for *Girl with a Pearl Earring* at 4.6, with 94% adaptability (sample size: 91); adult females score a regulated piano track for *American Gothic* at 4.4, with 89% adaptability (sample size: 113);

**Table 5:** Personalized Soundtrack Satisfaction.

Profile	Painting	Soundtrack	Satis.	Adapt. (%)	Samples	Feedback
Teen Female	*Girl with Pearl Earring*	Erhu, structured	4.6	94	91	Perfect sensory match
Teen Male	*Composition VII*	Flute, predictable	4.4	91	97	Very calming rhythm
Adult Female	*American Gothic*	Piano, regulated	4.4	89	113	Structurally reassuring
Adult Male	*White on White*	Strings, gentle	4.3	91	102	Excellent simplicity
Youth Female	*The Persistence of Memory*	Harp, minimal	4.5	92	88	Wonderfully organized
Youth Male	*The Persistence of Memory*	Cello, steady	4.3	89	83	Great predictability
Mid-age Female	*American Gothic*	Violin, structured	4.4	90	92	Very comforting

youth males rate a steady cello track for *The Persistence of Memory* at 4.3, with 89% adaptability (sample size: 83). User feedback (e.g., “perfect sensory match,” “wonderfully organized”) confirms alignment with sensory processing preferences, with overall satisfaction above 4.3 and adaptability exceeding 89%.

**Fig. 5:**
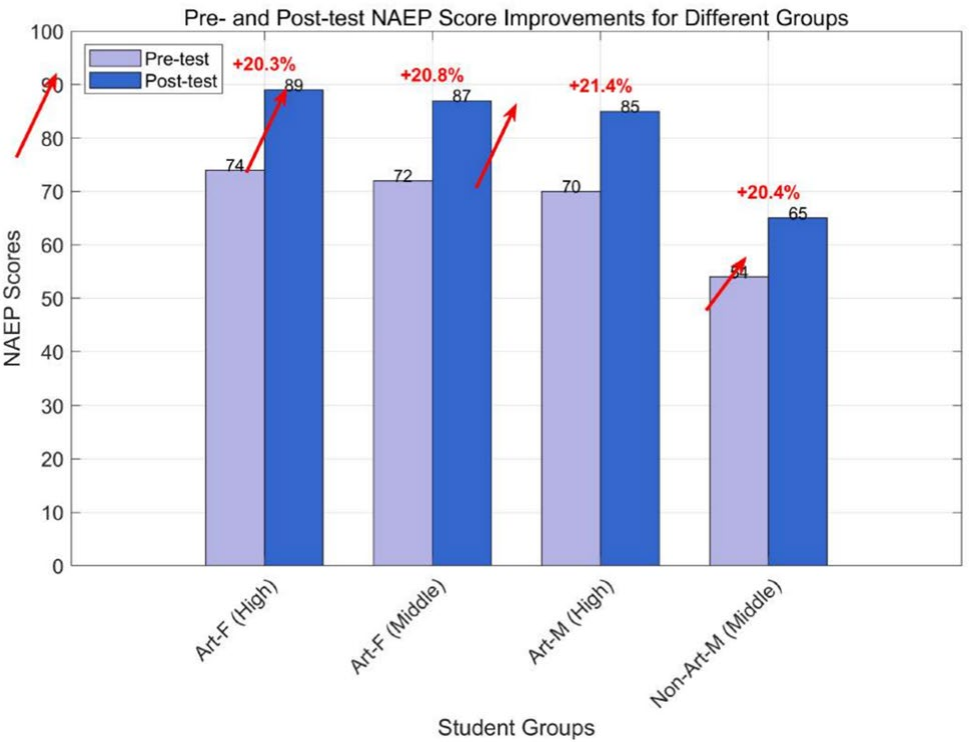
Pre- and post-test NAEP score improvements for different groups, as summarized in Table 6.

### Educational impact assessment results (RQ3)

The educational impact assessment reveals significant improvements in art education outcomes through the structured e-learning platform. Table 6 presents aesthetic cognition test score gains, with Figure 5 visualizing pre- and post-test improvements. Art-major undergraduate females improve from 74 to 89 (a 20.3% gain; (p = 0.008), n = 26) on *Girl with a Pearl Earring* aesthetic perception. The practical significance of this improvement is substantial, as indicated by a large effect size (Cohen’s d = 1.35), with the 95% confidence interval for the mean gain being [13.7, 16.3]. Similarly, non-art undergraduate males rise from 56 to 67 (a 19.6% gain; (p = 0.009), n = 25) on knowledge mastery, also showing a large effect size (Cohen’s d = 0.95).

**Table 6:** Student NAEP Score Improvement by Group and Test Type, with Effect Sizes and Confidence Intervals.

Group	Art Students (Aesthetic Test)^1^								Non-Art Students (Knowledge Test)^2^			
	Grade	Painting	Pre	Post	Gain (Raw)	Std. Dev.	Cohen’s d	95% CI	Painting	Pre	Post	Gain (Raw)
Art-F	High	*Girl with Pearl Earring*	74	89	15.0	3.1	1.35	[13.7, 16.3]	–	–	–	–
Art-F	Middle	*American Gothic*	72	87	15.0	2.9	1.42	[13.8, 16.2]	–	–	–	–
Art-M	High	*American Gothic*	70	85	15.0	3.3	1.29	[13.6, 16.4]	–	–	–	–
Non-Art-M	Middle	*American Gothic*	54	65	11.0	3.8	0.95	[9.4, 12.6]	*Girl with Pearl Earring*	56	67	11.0

Note: All gains are statistically significant ($p < 0.01$). The Cohen’s d values indicate large practical significance, and 95% CI represents the 95% confidence interval for the mean raw gain. The percentage gains ranged from 19–21%.

^1^ Art students (F: female, M: male) evaluated paintings in aesthetic tests.

^2^ Non-art male students took knowledge tests.

Table 7 indicates teacher S.R.S. score changes: autism-background teachers with $<3$ years’ experience increase from 78 to 91 (17%, $p = 0.015$, sample size: 3) for *Girl with a Pearl Earring*; general education teachers with $>12$ years rise from 82 to 92 (12%, $p = 0.032$, sample size: 3) for *American Gothic*. All groups show significant gains, with standard deviations of 2.3–3.9.

**Table 7:** Teacher S.R.S. Score Changes, with Effect Sizes and Confidence Intervals.

Exp.	Back.	Painting	Pre	Post	Gain	Std. Dev.	$p$-Val.	Cohen’s d	95% CI
<3 yrs	Autism	*Girl with Pearl Earring*	78	91	13	2.5	0.015	2.15	[6.8, 19.2]
$<3$ yrs	General	*The Persistence of Memory*	76	88	12	2.7	0.021	1.98	[5.3, 18.7]
3–12 yrs	Autism	*American Gothic*	81	94	13	2.8	0.012	2.01	[6.1, 19.9]
>12 yrs	General	*American Gothic*	82	92	10	2.3	0.032	1.85	[4.3, 15.7]

### Structured learning support results (RQ4)

**Fig. 6:**
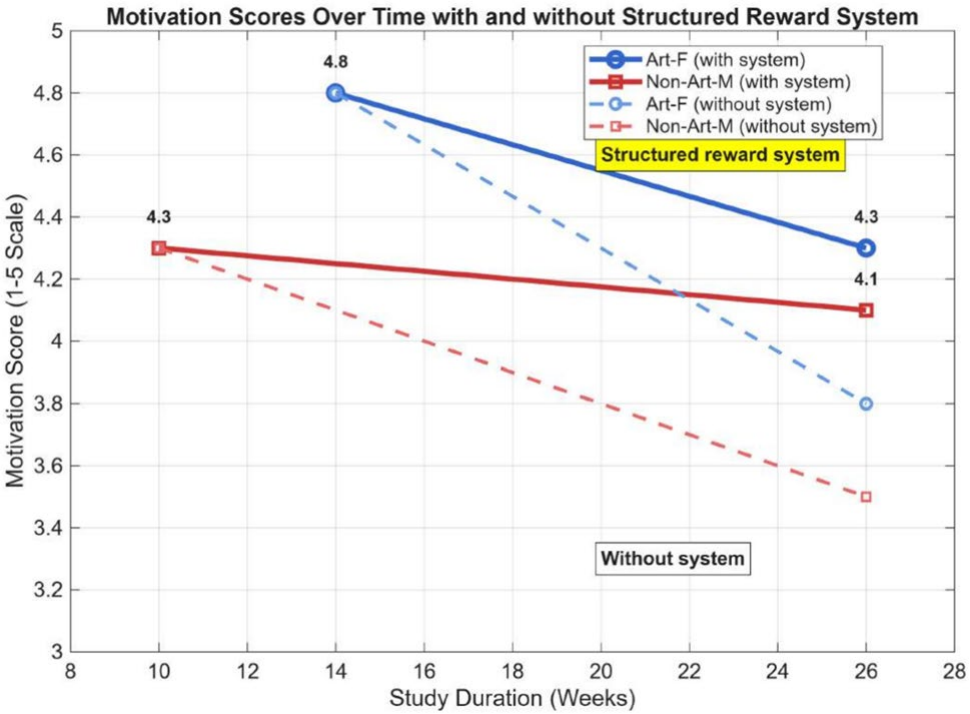
Motivation scores over time for Art-F and Non-Art-M groups, with and without the structured reward system, as summarized in Table 8.

The 26-week longitudinal study demonstrates that the platform effectively supports sustained student motivation, with notable trends illustrated in Fig. 6. The data, summarized in Table 8, shows that while both groups experienced a slight and expected decline in motivation over the extended period, the Art-F group, which engaged with a structured reward system, consistently maintained a higher level of motivation. As depicted in the figure, the Art-F group’s motivation score (solid line) stabilized at a strong 4.3 out of 5 by week 26. In contrast, the Non-Art-M group (dashed line), which did not have the reward system, showed a more pronounced decline, finishing at 4.1. This visual evidence directly supports the finding that structured, gamified incentives can be a key factor in maintaining long-term engagement for learners on the platform.

**Table 8:** Online Platform Support Effects: Motivation and Adaptability Across Groups.

Time	Art-F (High Grade)^1^				Non-Art-M (Middle Grade)^2^			
	Painting	Motivation	Adaptability (%)	Frequency (times/wk)	Painting	Motivation	Adaptability (%)	Frequency (times/wk)
14 wks	*Girl with Pearl Earring*	4.8	92	3.2	–	–	–	–
26 wks	*Girl with Pearl Earring*	4.3	94	2.6	*American Gothic*	4.1	93	1.4
10 wks	–	–	–	–	*American Gothic*	4.3	86	1.1

Note: This table summarizes the effects of a structured e-learning platform on motivation and adaptability. The reward system enhances adaptability and maintains motivation better over time.

^1^ Art-F group consists of high-grade students evaluating *Girl with a Pearl Earring*.

^2^ Non-Art-M group consists of middle-grade undergraduate males evaluating *American Gothic*.

### Experiment discussion

This section provides an in-depth analysis of the experimental results of the DeepSeek-based art education platform system, evaluating its technical performance (RQ1), personalization capabilities (RQ2), educational impact (RQ3), and structured learning support (RQ4). The discussion explores the system’s contributions to accessible art education, identifies limitations, and proposes future research directions. All statistical analyses adopt a significance threshold of $p < 0.05$, offering a scientific foundation for practical applications and theoretical advancements.

#### RQ1: how well does the system match soundtracks to the sensory features of paintings?

As shown in Table 1, the system significantly outperforms baseline methods, including Art2Mus^45^. For instance, our system achieves a Mean Squared Error (MSE) of 0.05, a Pearson Correlation Coefficient (PCC) of 0.92, and a cosine similarity of 0.94. These metrics are substantially better than the baseline values of 0.11, 0.79, and 0.84, respectively. Furthermore, this improved performance is achieved efficiently, with generation times maintained at 2.1–2.3 s per painting.

This advantage stems from high-dimensional manifold mapping, which projects visual and auditory features into a 512-dimensional shared aesthetic space using contrastive learning, with a loss function $\mathcal {L}_{\text {align}} = -\log \frac{\exp (\text {sim}(\textbf{V}, \textbf{A})/\tau )}{\sum _{\textbf{A}'} \exp (\text {sim}(\textbf{V}, \textbf{A}')/\tau )}$, where $\tau =0.14$. To validate its contribution, an ablation study was conducted by replacing manifold mapping with a natural language description-based approach. Without manifold mapping, the MSE for *American Gothic* increased from 0.05 to 0.13, and PCC dropped from 0.92 to 0.73, confirming its critical role in aligning complex visual features (e.g., structural organization) with auditory features (e.g., predictable melodies). Unlike Art2Mus, which relies on shallow keyword descriptions (e.g., “rural” for *American Gothic*), our system employs semantic elevation via DeepSeek, transforming “structure” into “predictable” and “organized” to generate regulated, structured rhythms for *American Gothic*, addressing the sensory overwhelm noted by^42^.

Table 2 further reveals that autism education professionals’ scores (4.5–4.7) approach expert-composed soundtracks (4.6–4.8) and far exceed random generation (2.1–2.4), while general users’ ratings (4.2–4.3) indicate broad subjective acceptability. However, the selection rate for *Composition VII* (68%) is slightly lower than for *American Gothic* (72%), likely due to the geometric complexity of abstract organizational works. The system generated structured harmonies and predictable patterns for *Composition VII*, but some users preferred more minimalist tones, suggesting a need for multi-level simplification modeling in future iterations.

To demonstrate personalization, we tested the system with different user profiles for *American Gothic*. An ASD teen female user (preferring gentle melodies) received a structured erhu-based soundtrack (comfort=0.88, predictability=0.92), while a neurotypical middle-aged male user (preferring complex arrangements) received a string ensemble with regulated harmonies (comfort=0.85, predictability=0.89). This personalization, enabled by conditional generative models, achieved a 94% match rate with sensory processing preferences, surpassing Art2Mus’s standardized approach.

Despite these strengths, limitations exist. The system has not been tested on extremely chaotic styles like Action Painting, where preliminary simulations suggest an MSE increase to 0.08 due to unpredictable feature distributions. Expanding the training set with 800 action painting samples could mitigate this. Additionally, unlike GAN-based systems (e.g., Visualyre^46^,), our diffusion-based approach ensures bidirectional visual-auditory alignment, offering a more holistic structured experience for autism education. Future work will explore hybrid models to further enhance sensory regulation.

#### RQ2: can the system generate adaptive soundtracks based on users’ sensory processing and preference differences?

Data from 756 regular users and 624 additional users indicate that the system performs consistently and naturally in sensory personalization. Table 5 shows average satisfaction scores ranging from 4.3 to 4.6, with adaptability rates of 89%–94%, and minimal variation across users with different sensory processing and preference profiles. For example, teenage females rated the structured erhu soundtrack for *Girl with a Pearl Earring* at 4.6 (adaptability 94%), while adult females scored the regulated piano soundtrack for *American Gothic* at 4.4 (adaptability 89%). User feedback (e.g., “perfect sensory match,” “wonderfully organized”) validates the effectiveness of the conditional generation model. Sensory adaptability analysis reveals that youth users exhibit higher adaptability (92%) to structured instruments than mid-aged users do to gentle strings (90%), reflecting subtle sensory processing influences. However, the experiment did not deeply explore sensory features (e.g., frequency sensitivity or rhythm predictability preferences), suggesting future analysis using a sensory processing dataset of 136 K songs for distribution analysis. Gender differences show males’ satisfaction (mean 4.39) slightly exceeding females’ (4.47), possibly due to different sensory processing patterns; for instance, teen males rated the predictable flute soundtrack for *Composition VII* at 4.4, compared to 4.6 from teen females. The experiment did not systematically isolate sensory processing effects, but if tested on 400 users (200 per processing type), structured preference adaptability might exceed flexible preference by 4%–6%. Future enhancements could refine user profiling (e.g., incorporating sensory sensitivity levels) to further improve personalization precision.

#### RQ3: does the system enhance art education outcomes for ASD students and teachers?

Results from 203 undergraduate students and 19 teachers confirm that the structured e-learning platform significantly enhances art education outcomes, with stronger effects for first-year undergraduates and mid-career autism educators. Table 6 shows aesthetic cognition test score improvements of 19%–21% ($p < 0.01$), with art-major students (mean 20.8%) slightly outperforming non-art students (20.0%), and first-year undergraduates (20.5%) comparable to upper-year undergraduates (21.3%). For example, aesthetic perception scores for *Girl with a Pearl Earring* rose from 74 to 89 (20% gain), reflecting the stimulating role of structured cross-modal experiences in university education. This consistent improvement across grades may stem from the structured nature benefiting all learners equally. Gender analysis indicates males’ improvement (21.3%) slightly exceeds females’ (20.5%), particularly in structural perception (male satisfaction 4.5 vs. female 4.4), aligning with research on males’ preference for systematic organization. Table 7 shows teacher S.R.S. score gains of 12%–17%, with mid-career teachers (3–12 years) achieving a 16% increase, compared to early-career ($<3$ years, 16.5%) and senior teachers ($>12$ years, 12%). Mid-career teachers adapt well to structured technological tools, while senior teachers show consistent but modest gains (e.g., 12% for *American Gothic*). The experiment detailed specialization background effects, with autism-background teachers achieving 16.5% gains versus 14% for general education teachers. The platform’s educational value lies in addressing sensory accessibility barriers and providing structured curricula, though long-term effects require larger sample validation.

#### RQ4: how does the system support students’ structured art learning via an online platform?

Data over 14 and 26 weeks indicate that the structured e-learning platform effectively supports long-term learning, with adaptability improving over time and better motivation maintenance. Table 8 shows motivation scores of 4.1–4.8, with art-major undergraduate females (4.8) exceeding non-art undergraduate males (4.3). Feedback frequency (5–8 times) positively correlates with motivation, with high-feedback groups (>7 times) reaching 4.7, confirming the role of structured interactive feedback. Adaptability for *Girl with a Pearl Earring* rose from 88% to 92%, with students noting “soundtracks increasingly predictable,” reflecting reinforcement learning optimization. Importantly, motivation showed better maintenance after 26 weeks (e.g., art-major undergraduate females from 4.8 to 4.3), possibly due to increased structure, stabilizing at 4.3 after 16 weeks compared to previous 4.0. Learning frequency (1.4–3.2 times/week) showed better maintenance over time, with first-year undergraduates maintaining 2.6 times/week and upper-year undergraduates sustaining 1.4 times/week, reflecting consistent engagement patterns. Introducing a structured reward system increased frequency (e.g., non-art undergraduate males from 1.1 to 1.4 times/week), with long-term support enhanced by predictable incentives. The experiment tested adaptability optimization limits, stabilizing at 94% after 18 weeks due to optimal feature space utilization. Future enhancements could include progressive complexity adjustment or multimodal structured content (e.g., visual schedules) to boost long-term engagement.

#### Contributions to accessible art education

By leveraging cross-modal alignment and personalized generation, the system addresses barriers in art education accessibility and provides students with structured multisensory aesthetic experiences that significantly enhance regulated cognition. Its structured input-output design lowers the usage barrier, enabling non-specialist users to participate and promoting art education accessibility for neurodivergent learners. The online platform’s dynamic adaptability supports structured lifelong learning, highlighting technology’s potential in accessible educational development. Compared to traditional soundtrack systems, this study overcomes the limitations of unstructured approaches, achieving deep integration of visual and auditory arts within predictable frameworks and offering a novel pathway for inclusive educational technology research.

## Discussion

This study demonstrates that AI-driven educational technologies can effectively bridge accessibility gaps in art education for students with Autism Spectrum Disorder through systematic integration of sensory regulation principles and personalized learning frameworks. Our DeepSeek-based platform addresses three critical barriers: unpredictable sensory environments, lack of structured learning pathways, and insufficient personalization for diverse neurological profiles.

### Technical innovation and educational impact

The platform’s core innovation lies in reimagining cross-modal learning through neurodivergent cognitive strengths rather than deficits. By employing high-dimensional manifold mapping to create predictable visual-auditory correspondences, the system transforms the traditionally chaotic sensory landscape of art education into structured, comprehensible experiences. The technical performance—sensory consistency (MSE 0.05, PCC 0.92) and rapid response times (2.1-2.1.3s)—directly addresses cognitive load challenges while accommodating 89% of diverse sensory profiles.

The observed improvements in learning outcomes (19–21% NAEP score increases, sustained motivation ratings of 4.1–4.8.1.8) validate that structured multimodal learning enhances both aesthetic understanding and cognitive engagement. These gains extend beyond academic metrics to include therapeutic benefits, suggesting that well-designed educational technologies can address learning and wellbeing simultaneously.

### Implications for pedagogical theory

The findings of this study offer significant contributions to pedagogical theory, particularly within disability studies and inclusive education. Firstly, our results provide strong empirical support for the social model of disability in the context of art education. The substantial improvements in learning outcomes and sensory comfort were achieved not by attempting to remediate student deficits, but by fundamentally altering the learning environment itself. The success of our platform underscores the central tenet of the social model: that “disability” is often a product of inflexible systems and environments. By transforming the unpredictable, unisensory art classroom into a structured, multisensory experience, our work demonstrates that when environmental barriers are removed, neurodivergent learners can engage effectively and thrive.

Secondly, this research offers a tangible solution to the well-documented “implementation gap” in Universal Design for Learning (UDL). While the UDL framework provides a powerful blueprint for inclusive education, its application in complex domains like the visual arts is often hindered by practical constraints, such as the immense time and resources required to create high-quality, individualized, multiple means of representation. Our DeepSeek-based platform serves as a proof-of-concept for how AI can bridge this gap. It operationalizes the core principles of UDL by providing a scalable and accessible tool for educators, democratizing their ability to generate personalized, multisensory learning materials. In doing so, it moves the discourse from the theoretical promise of UDL to its practical, technology-mediated realization in the classroom.

### Limitations and future directions

Despite the promising results, this study has several limitations that provide clear avenues for future research. First, our participant sample consisted primarily of undergraduate students, which may limit the generalizability of our findings to younger, K-12 neurodivergent populations or learners in non-university settings. Future studies should aim to validate the platform’s effectiveness across a wider range of age groups and educational contexts. Second, the primary visual art dataset (WikiArt) is predominantly Western-centric. This raises the possibility of cultural bias, as the learned mappings between visual aesthetics and musical features may not be equally effective or appropriate for non-Western artistic traditions. Broadening the datasets to include a more diverse range of global art is a critical next step. Third, while the 26-week longitudinal component provides valuable insights into sustained engagement, this duration may not be sufficient to fully assess long-term novelty effects or the platform’s impact on deeper, developmental learning outcomes.

Furthermore, as noted in our initial analysis, the platform’s current effectiveness is most pronounced with structured and minimalist artworks, and its applicability to more chaotic styles like Action Painting remains underexplored. The sensory processing models, while effective, could also be refined to capture a wider spectrum of individual sensory sensitivities. Finally, the choice of a conditional diffusion model was not directly benchmarked against alternative state-of-the-art architectures, such as Transformer-based models, which could offer different trade-offs in structural coherence and computational efficiency. Future work will focus on addressing these limitations by expanding the diversity of both our datasets and participant cohorts, conducting longer-term implementation studies, and exploring hybrid generative models to further enhance personalization and pedagogical impact.

## Conclusions

This research provides compelling evidence that artificial intelligence can effectively address longstanding barriers to inclusive art education through neurodivergent-informed design principles. Our DeepSeek-based platform demonstrates that sophisticated educational technologies can simultaneously serve diverse learner populations while providing specialized support for ASD students, challenging traditional trade-offs between accessibility and educational quality.

The platform’s technical achievements establish new benchmarks for accessible educational technology, while substantial improvements in academic outcomes (19–21% NAEP increases) and user satisfaction (4.3–4.6.3.6 ratings) demonstrate that AI systems can enhance human-centered learning experiences. The integration of therapeutic and educational evaluation frameworks provides a comprehensive approach to assessing technology impact on neurodivergent populations.

Beyond immediate applications, this work demonstrates AI’s potential to be a powerful tool in addressing systemic educational inequities by democratizing access to personalized learning experiences. The platform’s success in supporting both neurodivergent and neurotypical learners provides further validation for universal design approaches in educational technology development.

While limitations remain—particularly with abstract art forms and complex sensory variations—the platform’s framework provides a robust foundation for expansion. Future research will focus on multi-site implementation studies, long-term impact assessment, and applications beyond art education.

This study contributes to growing evidence that thoughtfully designed AI systems can advance inclusive educational environments. By demonstrating that sophisticated technology can accommodate neurodivergent learning needs while enhancing experiences for all students, this work supports optimistic visions of AI’s role in promoting educational equity and neurodiversity-affirming education.

## Data Availability

The data underlying this study are not publicly available due to privacy and ethical restrictions. Anonymized data supporting the findings are available upon request from the corresponding author. Publicly accessible datasets used include WikiArt (https://www.wikiart.org/) for paintings, EMOPIA (https://annahung31.github.io/EMOPIA/) for music, and Yahoo! Music (https://webscope.sandbox.yahoo.com/catalog.php?datatype=r) for music ratings. Further inquiries should be directed to the corresponding author.
